# A survey for piroplasmids in questing *Ixodes fuscipes* ticks reveals undescribed *Babesia* lineages in Uruguay

**DOI:** 10.1186/s13071-025-06866-0

**Published:** 2025-06-18

**Authors:** Rodrigo Alvez, María L. Félix, Adriana Santodomingo, Pablo Parodi, Richard Thomas, Sebastián Muñoz-Leal, Luis Carvalho, José M. Venzal

**Affiliations:** 1 Laboratorio de Vectores y Enfermedades Transmitidas, Departamento de Ciencias Biológicas, CENUR Litoral Norte, Universidad de la República, Salto, Uruguay; 2 Centro de Investigación de Estudios Avanzados del Maule (CIEAM), Vicerrectoría de Investigación y Postgrado, Universidad Católica del Maule, Talca, Chile; 3 Instituto Nacional de Investigación Agropecuaria, Plataforma de Investigación en Salud Animal, Estación Experimental INIA Tacuarembó, Tacuarembó, Uruguay; 4 Departamento de Ciencia Animal, Facultad de Ciencias Veterinarias, Universidad de Concepción, Chillán, Ñuble Chile; 5 AgResearch, Grasslands Research Centre, Palmerston North, New Zealand; 6 PEDECIBA, Programa de Desarrollo de las Ciencias Básicas, Montevideo, Uruguay

**Keywords:** Vector-borne piroplasmids, *Babesia* spp., *Ixodes fuscipes*, One Health, Uruguay

## Abstract

**Background:**

*Ixodes fuscipes* is a tick species found in the Southern Cone of America and the only member of the *Ixodes ricinus* complex present in Uruguay. Members of this complex are particularly recognized as vectors of diseases affecting human health, such as babesiosis, caused by parasites of the genus *Babesia* (Apicomplexa: Piroplasmida). However, even though potential hosts of *I. fuscipes* in Uruguay (rodents, birds, and artiodactyls) are known carriers of *Babesia* species, the potential role of *I. fuscipes* as a vector of piroplasmids has not been studied.

**Methods:**

In this study, questing *I. fuscipes* ticks were collected from five locations in Uruguay, and the presence of piroplasmid DNA was assessed using polymerase chain reaction (PCR) to amplify fragments of the small subunit ribosomal RNA (*18S* rRNA) and cytochrome c oxidase subunit 1 (*COI*) genes.

**Results:**

A total of 953 ticks (larvae, nymphs, and adults) were collected; 14 samples (two larval pools and 12 nymphs) tested positive. Genetic analyses using *18S* rDNA and *COI* sequences revealed the presence of undescribed *Babesia* lineages, belonging to the *Babesia odocoilei* clade and others to the *Babesia microti* sensu stricto clade.

**Conclusions:**

This work represents the first association of *Babesia* spp. with *I. fuscipes* and highlights the importance of this type of study to detect and mitigate the emergence of diseases associated with these arthropods.

**Graphical Abstract:**

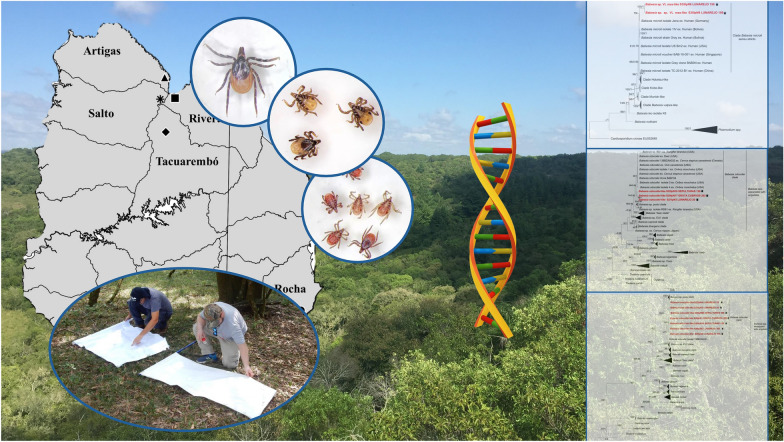

**Supplementary Information:**

The online version contains supplementary material available at 10.1186/s13071-025-06866-0.

## Background

The genus *Ixodes* (Acari: Ixodidae) is considered the most ancient and diverse tick lineage, with over 280 recognized species worldwide [1–4]. *Ixodes* ticks primarily exploit passerine birds, rodents, carnivores, and ungulates; however, some species also infest squamates [5, 6].

Among the *Ixodes* spp., members of the *Ixodes ricinus* complex are particularly notable as vectors of bacteria and protozoa causing significant human diseases, such as Lyme borreliosis (caused by *Borrelia burgdorferi*, *Borrelia afzelii*, *Borrelia garinii*, and others); human granulocytic anaplasmosis (by *Anaplasma phagocytophilum*); and babesiosis (by *Babesia* spp.) [7, 8]. The *I. ricinus* complex, defined by Keirans et al. [9], currently comprises ~20 species, three of which (*Ixodes pararicinus*, *Ixodes fuscipes*, and *Ixodes chacoensis*) occur in the Southern Cone of America [3, 5, 10].

Babesiosis, caused by parasites of the genus *Babesia* (Apicomplexa: Piroplasmida), is an emerging malaria-like disease with growing global relevance owing to its impact on human health, livestock, and pets [11]. Piroplasmids of the genus *Babesia* are obligate intracellular parasites that exhibit complex life cycles alternating between vertebrate hosts and ticks, where they reproduce asexually in erythrocytes and sexually within tick cells [12]. Unlike *Babesia*, which exclusively infect erythrocytes throughout its life cycle, other members of the order Piroplasmida, such as *Cytauxzoon* and *Theileria*, invade leukocytes during their early developmental stages [12, 13].

Recent advances in the molecular systematics of the order Piroplasmida, incorporating datasets of long *18S* rRNA gene sequences, have delineated four major clades within the genus *Babesia*, each with distinct evolutionary trajectories and host specificities: the *Babesia* sensu stricto (s.s.), Western *Babesia*, *Babesia microti*-like, and *peircei* groups [12, 13]. Particularly, the *B*. *microti*-like group represents a medically significant complex of species in the northern latitudes, which includes five minor clades: *B*. *microti* s.s. (clade 1); *Babesia vulpes*-like (clade 2); Munich-like (clade 3); Kobe-like (clade 4); and Hobetsu-like (clade 5) [12, 14, 15].

The *Babesia* s.s. clade comprises species that predominantly infect ungulates and are characterized by their ability to undergo both transstadial (through tick developmental stages) and transovarial (vertical) transmission [12]. Traits that allow their long-term persistence in tick populations and vertebrate hosts. This group includes economically and clinically important species, such as *Babesia bovis*, *Babesia vulpes*, *Babesia divergens*, *Babesia odocoilei*, *Babesia venatorum*, and *Babesia crassa*-like [12, 13, 16–19]. In contrast, lineages within the *B. microti-*like group, such as *B. microti* s.s., primarily infect rodents and depend exclusively on transstadial transmission for their survival in tick vectors [12, 16]. Other transmission routes in *Babesia*, such as blood transfusion, and organ transplantation, have also been documented [12].

Human babesiosis has increased in prevalence globally, with zoonotic lineages within the *Babesia* s.s. (*B. divergens*, *B. odocoilei*, *B. venatorum*, *B. crassa-*like organisms) and *B. microti*-like groups being key causative agents [12, 19, 20]. In South America, *Babesia* lineages related to *B*. *odocoilei* and the *B*. *microti*-like group have recently been identified in wild hosts and *Ixodes* ticks, shedding light on their epidemiology and systematics beyond the Northern Hemisphere [21–23].

In Uruguay, however, research on piroplasmid infections has largely focused on domestic animals [24–26], neglecting wildlife reservoirs and their potential vectors [27, 28]. Of the four *Ixodes* species reported in the country: *Ixodes auritulus*, *Ixodes fuscipes*, *Ixodes longiscutatus*, and *Ixodes loricatus*, only *I. fuscipes* belongs to the *I. ricinus* complex, yet no piroplasmids have been associated with it despite its potential as a vector [5]. The vertebrate host profile of *I. fuscipes* is diverse, with adults often feeding on artiodactyls and immature stages parasitizing rodents and birds [6, 29]. Notably, artiodactyls and rodents are well-documented reservoirs for *Babesia* spp. associated with ungulates and the *B. microti*-like group, respectively [12, 17, 30, 31]. Increasing evidence for possible zoonotic *Babesia* lineages in the native wildlife of South America [21–23] underscores the need to explore the role of *I*. *fuscipes* as a potential host for piroplasmids.

In this study, questing *I. fuscipes* ticks collected in Uruguay were screened for Piroplasmida DNA to address the identified knowledge gaps. We implemented molecular analyses targeting the small subunit ribosomal RNA gene (*18S* rRNA) and mitochondrial markers, including cytochrome c oxidase subunit 1 (*COI*) and cytochrome *b* (*cytb*), which have proven effective for resolving deep phylogenetic relationships (*18S* rRNA) and delineating species (*COI* and *cytb*) within the Piroplasmida order [12–17, 32].

## Methods

### Study area

**Fig. 1:**
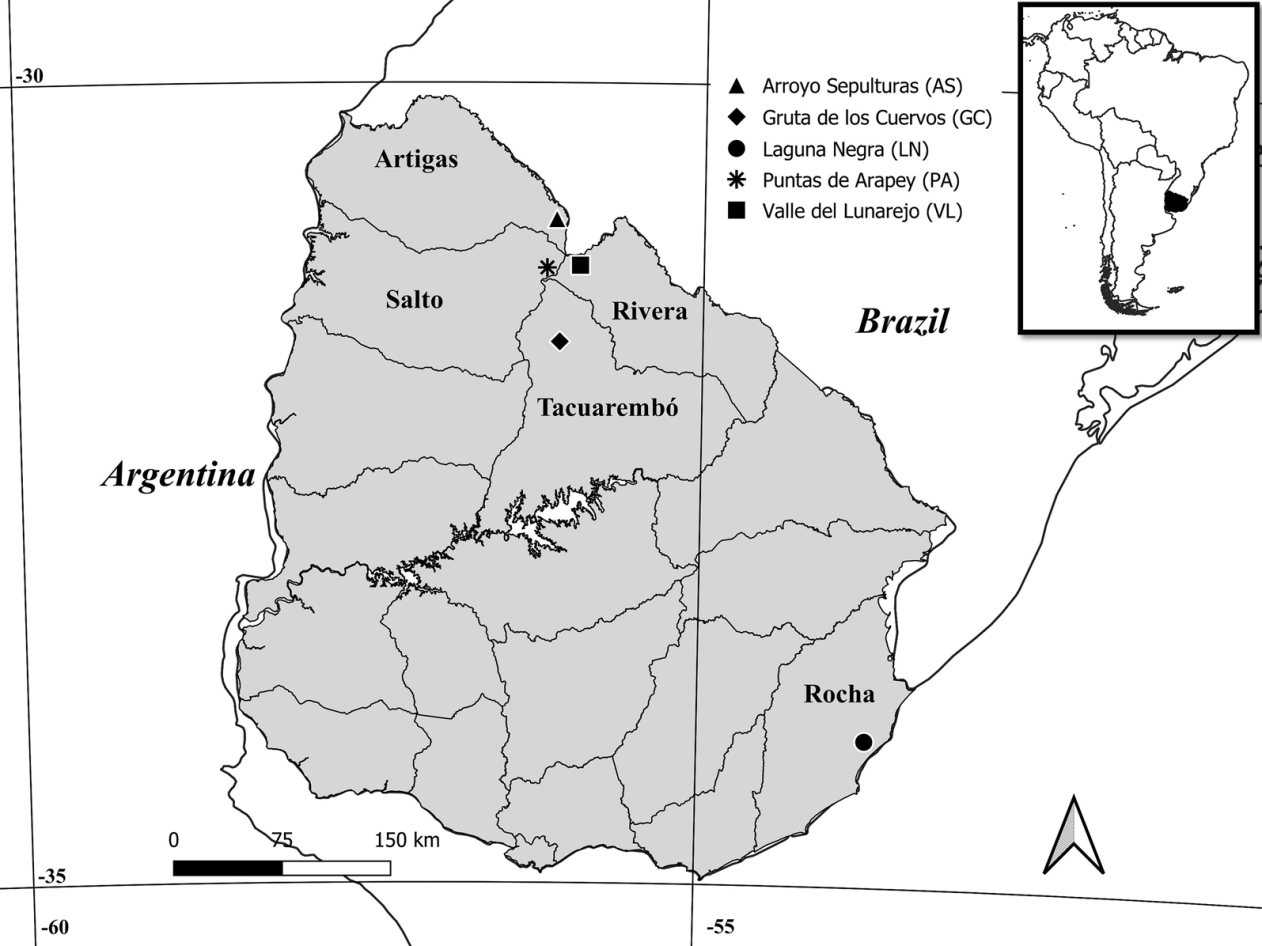
Map of Uruguay showing the locations where ticks were collected (developed using the open-source Geographic Information Systems QGIS 3.32.3)

Field sampling was conducted at five sites across Uruguay between September 2017 and May 2023 (Fig. 1). A total of 22 collection trips were performed during this period, encompassing all seasons of the year.

Four sites are located in the north of the country: Arroyo Sepulturas (AS), in Artigas Department (−30.851389, −56.072500); Valle del Lunarejo (VL), in Rivera Department (−31.141389, −55.900278); Puntas de Arapey (PA), in Salto Department (−31.156944, −56.140556); and Gruta de los Cuervos (GC), in Tacuarembó Department (−31.618889, −56.046389). The fifth site, Laguna Negra (LN), is situated in the southern region, in Rocha Department (−34.085833, −53.738056).

### Tick collection and identification

Questing ticks were collected from the vegetation using the flagging method, which involves systematically dragging a 1.20 m × 0.80 m white cloth across the vegetation and inspecting it for attached ticks at regular intervals of 5–10 m. The collected ticks were preserved in tubes containing absolute ethanol (Sigma-Aldrich^®^) for subsequent morphological identification and molecular analyses [33–35].

Ticks were identified on the basis of their morphological features using taxonomic keys provided by Venzal et al. [36] and Nava et al. [5], and a Nikon SMZ1000 stereo microscope (Nikon Corporation, Tokio, Japan). To further validate tick species, a fragment of the mitochondrial 16S ribosomal rRNA (16S rRNA) gene was sequenced [37].

### DNA isolation

Adult ticks were bisected longitudinally using sterile scalpel blades. One half was used for genomic DNA extraction, while the other was preserved in absolute ethanol. Nymphs were dissected and processed individually, and larvae were pooled by collection site and date, with no more than ten specimens per pool.

Genomic DNA was extracted using the GeneJET Genomic DNA Purification kit (Thermo Scientific, Vilnius, Lithuania) according to the manufacturer’s instructions and eluted in 100 μL of buffer (10 mM Tris hydrochloride (Tris–Cl), pH 9.0, 0.1 mM ethylenediaminetetraacetic acid (EDTA)). The DNA concentration was then quantified with a Nanodrop 2000 spectrophotometer (Thermo Fisher Scientific, Waltham, MA, USA), and the DNA quality was evaluated on the basis of the A260/A280 ratio. Samples with values between 1.6 and 2.0 were deemed suitable for polymerase chain reaction (PCR) amplifications protocols [38].

### Gene amplification and sequencing

An initial PCR screening was conducted to detect piroplasm DNA, targeting a ~551-base pair (bp) fragment of the *18S* rRNA gene [39]. Samples that tested positive in the screening PCR underwent a second PCR, which amplified a ~1500-bp fragment of the same gene [40]. In addition, two other PCRs were performed: one amplifying a ~1080-bp fragment of the *COI* gene and another a ~1300-bp fragment of the *cytb* gene [16]. Primers and PCR thermal cycling conditions used in this study are detailed in Table 1.

**Table 1:** Primers and thermal conditions used for PCR detection and genetic characterization of ticks and Piroplasmid

Organism	Gene	Primer	Sequence	*T*_m_ (°C)	Expected length (bp)	References
Ticks	16S rRNA	16S + 1	CCGGTCTCAACTCAGATCAAGT	*47–48.8 phase 1, 50 phase 2	460	[37]
		16S + 1	GCTCAATGATTTTTTAAATTGCTGT			
Piroplasmida	*18S* rRNA	BAB2-F BAB2-R	CCGTGCTAATTGTAGGGCTAATACA GCTTGAAACACTCTARTTTTCTCAAAG	58	551	[39]
	*18S* rRNA	Nbab_1F	AAGCCATGCATGTCTAAGTATAAGCTTTT	60	1500	[21, 40, 86]
		18SApiR	GGATCACTCGATCGGTAGGAG			
	*COI*	COI-F	GGAAGTGGWACWGGWTGGAC	59–60	1080	[16, 21]
		COI-R	TTCGGTATTGCATGCCTTG			
	*cytb*	cytb-F	TTAGTGAAGGAACTTGACAGGT	55	1300	[16, 21]
		cytb-R	CGGTTAATCTTTCCTATTCCTTACG			

*The annealing temperature of the first 7 cycles in phase 1 was increased by 0.3 °C every second cycle from 47 to 48.8 °C, followed by 28 cycles using an annealing temperature of 50 °C in phase 2

PCRs were performed in a SimpliAmp™ thermal cycler (Applied Biosystem, Waltham, MA, USA) in a 25 μL reaction mixture, containing 2 μL of each primer (0.4 μM), 4.5 μL of ultrapure water, 12.5 μL of MangoMix (Bioline, Memphis, TN, USA), and 4 μL of template DNA. *Babesia bovis* isolate Uy_Bbo (GenBank accession no. PQ272560) DNA was used as a positive control, and nuclease-free water as the negative control. PCR products were stained with GoodView Nucleic Acid Stain (Beijing SBS Genetech Co. Ltd., China), separated by electrophoresis on 1.5% agarose gel, and visualized using a CSLUVTSDUO312 ultraviolet (UV) transilluminator (Cleaver Scientific Ltd., UK). Amplicons of the expected size were purified using a GeneJET PCR purification Kit (Thermo Fisher Scientific, Vilnius, Lithuania) and then submitted for bidirectional Sanger sequencing at Macrogen (Seoul, South Korea).

### Assembly and sequence analyses

Sanger sequencing data were visualized, quality-checked, and edited with Geneious Prime® version 2024.0.3 (www.geneious.com) to generate consensus sequences. Base calls with Phred values ≥ 20 were considered adequate for further analyses [41, 42]. Consensus sequences underwent pairwise comparisons using the BLASTn tool (https://blast.ncbi.nlm.nih.gov/Blast.cgi) to identify orthologous sequences.

Sequences obtained in this study, along with orthologous sequences retrieved from the GenBank (https://www.ncbi.nlm.nih.gov/nuccore), were used to construct multiple sequence alignments using the multiple sequence alignment program (MAFFT) tool with default settings [43]. To map informative regions for phylogenies, we curated the alignments using the Block Mapping and Gathering with Entropy (BMGE) tool using predetermined parameters [44].

### Phylogenetic analyses

In molecular systematics, phylogenetic inferences rely on the character states of terminal taxa, represented by their sequences [45, 46]. While the primary focus lies in understanding the relationships among ingroup sequences, including outgroup sequences provides a critical reference for character state polarization and for testing hypotheses about the evolutionary relationships within the ingroup [47, 48]. Furthermore, phylogenetic inferences based on sequences from a wide range of taxa have demonstrated that incorporating denser sequence sampling improves the resolution and support of internal nodes, enhances confidence in character state polarization, and mitigates phylogenetic artifacts such as long-branch attraction [23, 49–56]. Following these principles, we implemented the phylogenetic frameworks proposed by Santodomingo et al. [21, 23] to analyze our sequences, ensuring robust and reliable phylogenetic inferences.

However, to address the heterogeneity in DNA sequence evolution, such as variability in substitution rates among sites, differences in base composition, and selective pressures, including codon evolution, we conducted phylogenies using maximum likelihood (ML) [45] and Bayesian inference (BI) [46, 57]. Both methods employ probabilistic models of DNA sequence evolution to account for these complexities, ensuring robust and accurate phylogenetic inference.

ML analyses were performed using IQ-TREE version 1.6.12 [58], while BI was conducted in MrBayes version 3.2.6 [59]. Given the heterogeneity in nucleotide exchange rates at the first, second, and third codon positions of protein-coding genes [59, 60], the *COI* dataset was partitioned into three codon positions: position-1, position-2, and position-3 [59–62].

For ML phylogenies, the ModelFinder command “TESTNEWONLY -mrate G” was implemented to select the best-fit evolutionary nucleotide models [63] for *18S* rRNA. Meanwhile, the command “-m TESTNEWONLYMERGE -mrate G” was used to estimate the best-fit evolutionary nucleotide models and the optimal partition scheme [63] for *COI*. The robustness of the inferred trees was assessed using a combination of hill-climbing approaches and a stochastic disturbance method, complemented by an ultrafast bootstrap (UFBoot) approach with 1000 iterations [58, 64]. UFBoot values of less than 70%, from 70 to 94%, and 95% or greater indicated nonsignificant, moderate, and high statistical support, respectively [64]. 

BI phylogenies were inferred on the basis of nucleotide substitution models computed with the MrBayes command “lset nst = mixed rates = gamma” and “lset nst = mixed rates = invgamma” [59, 65] for *18S* rRNA and *COI*, respectively. Partition schemes for BI phylogenies were set according to schemes determined by ModelFinder [61, 63]. The robustness of the inferred trees was evaluated by sampling trees every 1000 generations, discarding the first 25% as burn-in, and implementing four Markov Chain Monte Carlo (MCMC) chains through two independent tests of 20 × 10^6^ generations [59]. Tracer version 1.7.1 [66] was used to assess the convergence and to confirm the effective sample size (ESS) of the MCMCs. Bayesian posterior probabilities (BPP) ≥ 0.71 in nodes, were considered high statistical support [67].

All best-fit nucleotide models and partitions schemes were selected on the basis of Bayesian information criteria [68]. Trees were visualized and edited with FigTree version 1.4.1 (http://tree.bio.ed.ac.uk/software/figtree/) and Inkscape version 1.1 (https://inkscape.org/es/). Consensus trees for both ML and BI were generated following the approach outline by Santodomingo et al. [21].

## Results

### Tick collection and identification

A total of 953 ticks were collected across all surveyed localities, comprising 608 larvae, 314 nymphs, 14 males, and 17 females. Larvae were grouped into 65 pools for further processing. The number of collected ticks by locality was as follows: 298 from AS, 453 from VL, two from PA, 184 from GC, and 16 from LN. A detailed breakdown of tick life stage per locality is presented in Table 2. All ticks were morphologically identified as *I*. *fuscipes*. PCRs targeting the mt 16S rRNA yielded amplicons of the expected size (~460 bp), validating the success of DNA extractions in all cases.

**Table 2:** Data for collected *Ixodes fuscipes* and detection of *Babesia* spp.

Locality of collection site (total ticks)	Stage	No. of ticks	Samples (individual or pools^*^)	Positive samples	Positive samples code	Date of collection positive samples	Species or genotypes	GenBank accession no.	
								*18S* ARNr	*COI*
Gruta de los Cuervos, Tacuarembó (184)	Larva	62	8^*^	0					
	Nymph	105	105	2	S39IpN17(202)	21 May 2022	*B. odocoilei*-like	PP512755	PP886515
					S40IpN9(276)	29 July 2022	*B. odocoilei*-like	PV061837	
	Male	9	9	0					
	Female	8	8	0					
Valle del Lunarejo, Rivera (453)	Larva	292	30^*^	1	S44IpL20(360)	12 May 2023	*B. odocoilei*-like	PV061838	
	Nymph	153	153	8	S21IpN3(20)	13 November 2017	*B. odocoilei*-like	PP512757	PP886520
					S22IpN4(29)	14 December 2017	*B. odocoilei*-like	PV061833	PP886519
					S35IpN6(156)	12 November 2021	*Babesia* sp. VL *microti* (*m*) s.s.-like	PP512753	
					S35IpN9(159)	12 November 2021	*Babesia* sp. VL *m*s.s.-like	PP512754	
					S36IpN5(165)	23 December 2021	*B. odocoilei*-like	PV061835	PP886517
					S36IpN6(166)	23 December 2021	*B. odocoilei*-like	PV098357	PP886516
					S40IpN3(270)	30 July 2022	*Babesia* sp. VL *m*s.s.-like	PV053416	
					S40IpN5(272)	30 July 2022	*B. odocoilei*-like	PV061836	
	Male	4	4	0					
	Female	4	4	0					
Arroyo Sepulturas, Artigas (298)	Larva	254	27^*^	1	S32IpL12(246)	7 July 2021	*B. odocoilei*-like	PV061834	
	Nymph	41	41	2	S32IpN18(128)	7 July 2021	*B. odocoilei*-like	PP512756	PP886518
					S44IpN66(406)	12 May 2023	*B. odocoilei*-like	PV061839	PP886514
	Female	3	3	0					
Puntas de Arapey, Salto (2)	Male	1	1	0					
	Female	1	1	0					
Laguna Negra, Rocha (16)	Nymph	15	15	0					
	Female	1	1	0					
Total		953	410	14					

Details of BLASTn sequence analysis, including percentage of identity, sequence size, gaps, query coverage, *E*-value, and GenBank accession numbers are presented in Supplementary Tables 1–4.

Mitochondrial 16S rRNA gene sequences generated from one tick per site [GenBank accession nos. PQ868241 (LN); PQ868242 (PA); PQ868243 (AS); PQ868244 (GC); PQ868245 (VL); and PQ868246 (VL)], after BLASTn analysis, showed 99.03–100% similarity to previously deposited *I. fuscipes* sequences from Brazil and Uruguay (Additional File 1: Supplementary Table S1), providing molecular support for the morphological identification. In addition, these sequences displayed similarities with other species within the *I. ricinus* complex native to the Southern Cone of America. Specifically, similarities ranged from 93.02 to 93.71% with *I. pararicnus* sequences, and from 93.38 to 94.33% with *I. chacoensis* sequences (Additional File 1: Supplementary Table S1).

### Piroplasmid detection and phylogenies

A total of 410 tick samples, including 65 larval pools and the remaining individual ticks (314 nymphs, 14 males, and 17 females), were analyzed for piroplasm detection. From this total, 14 samples (3.42%; two larval pools and 12 nymphs) from three of the five surveyed localities tested positive by PCR screening targeting the *18S* rRNA gene, yielding amplicons ranging from 486 to 551 bp. In addition, five of these samples produced longer *18S* rDNA sequences (1393–1475 bp). Only seven *COI* sequences were generated for mitochondrial markers, with lengths ranging from 891 to 1066 bp (Table 2). None of the samples produced amplicons for *cytb* gene. 

BLASTn analyses of 11 *18S* rDNA sequences (486–1415 bp) from larvae and nymphs collected at GC (2 sequences), VL (6 sequences), and AS (3 sequences) showed 98.75–99.58% similarity to *B*. *odocoilei* sequences previously characterized from cervids and ticks in Canada and the USA. Moreover, these sequences exhibited 99.06–99.64% similarity with *Babesia* sp. *pudui* sequences from Chile (Additional File 2: Supplementary Table S2). The remaining three *18S* rDNA sequences (523–1477 bp), derived from nymphs collected at VL, showed 96.95–98.44% similarity to *B. microti* sequences from various locations worldwide (Additional File 3: Supplementary Table S3).

For *COI* gene, comparisons revealed 97.36–97.60% identity with *B*. *odocoilei* sequences identified from western red deer (*Cervus elaphus*) in Canada and 93.15–93.72% identity with *Babesia* sp. *pudui* identified from southern pudu (*Pudu puda*) and *Ixodes stilesi* ticks in Chile (Additional File 4: Supplementary Table S4).

**Fig. 2:**
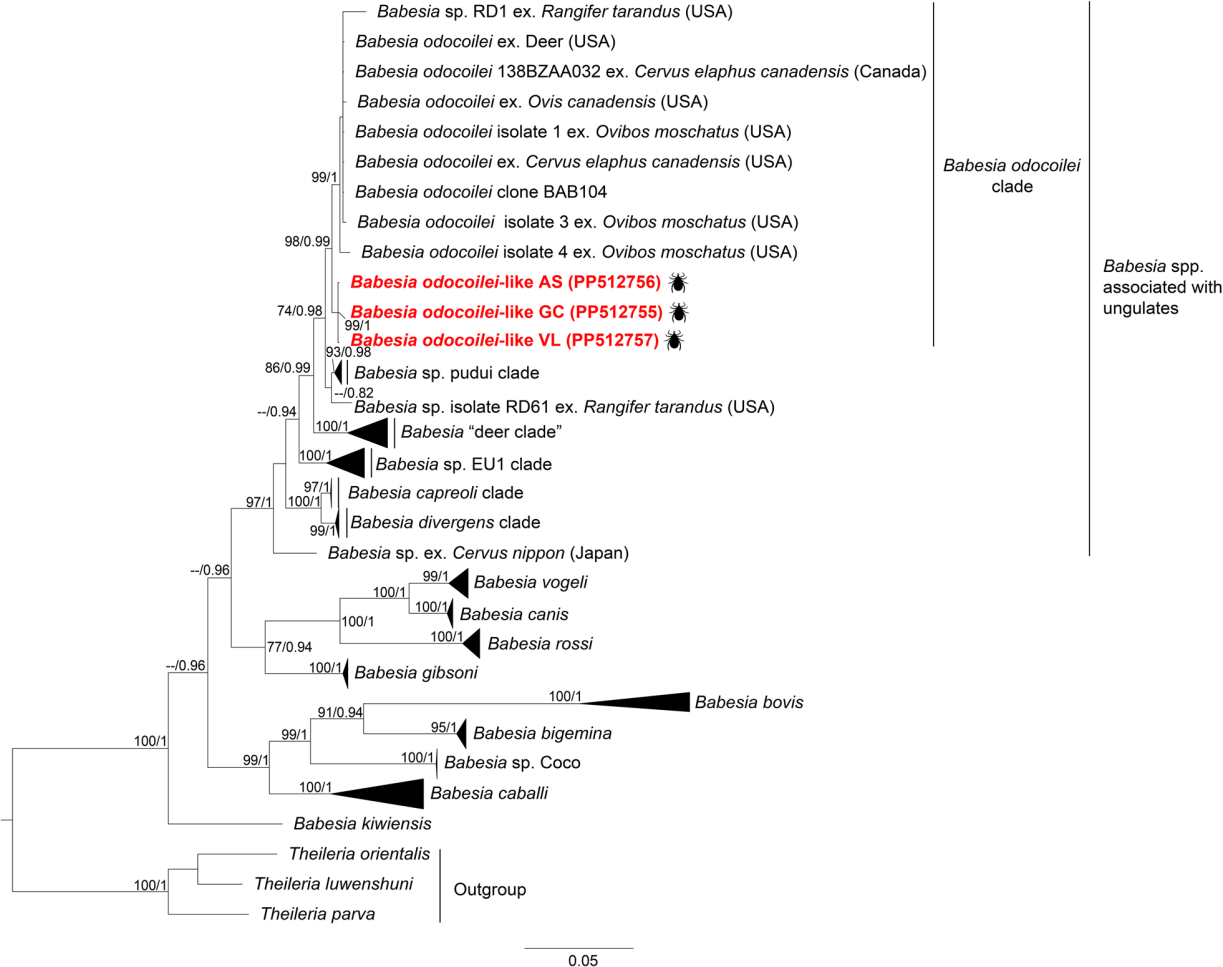
Maximum likelihood (ML) and Bayesian inference (BI) consensus trees of Piroplasmida, highlighting the phylogenetic placement of *Babesia odocoilei*-like (labeled in red font). Phylogenetic analyses were conducted incorporating 90 sequences for the *18S* rRNA gene (aligned for 1612 bp). The best-fit evolutionary ML and BI models were determined for each gene dataset. For the ML, the model was TIM2 + F + G4, while the BI models included *M*_94_, *M*_36_,* M*_70_,* M*_6_. Only nodes with ultrafast bootstrap values > 70% for ML [64] and Bayesian posterior probabilities ≥ 0.71 for BI [67] are shown above or below each branch. The scale bar represents the number of nucleotide substitutions per site. Details and GenBank accession numbers of sequences in collapse clades are provided in Additional File 5: Supplementary Table S5

**Fig. 3:**
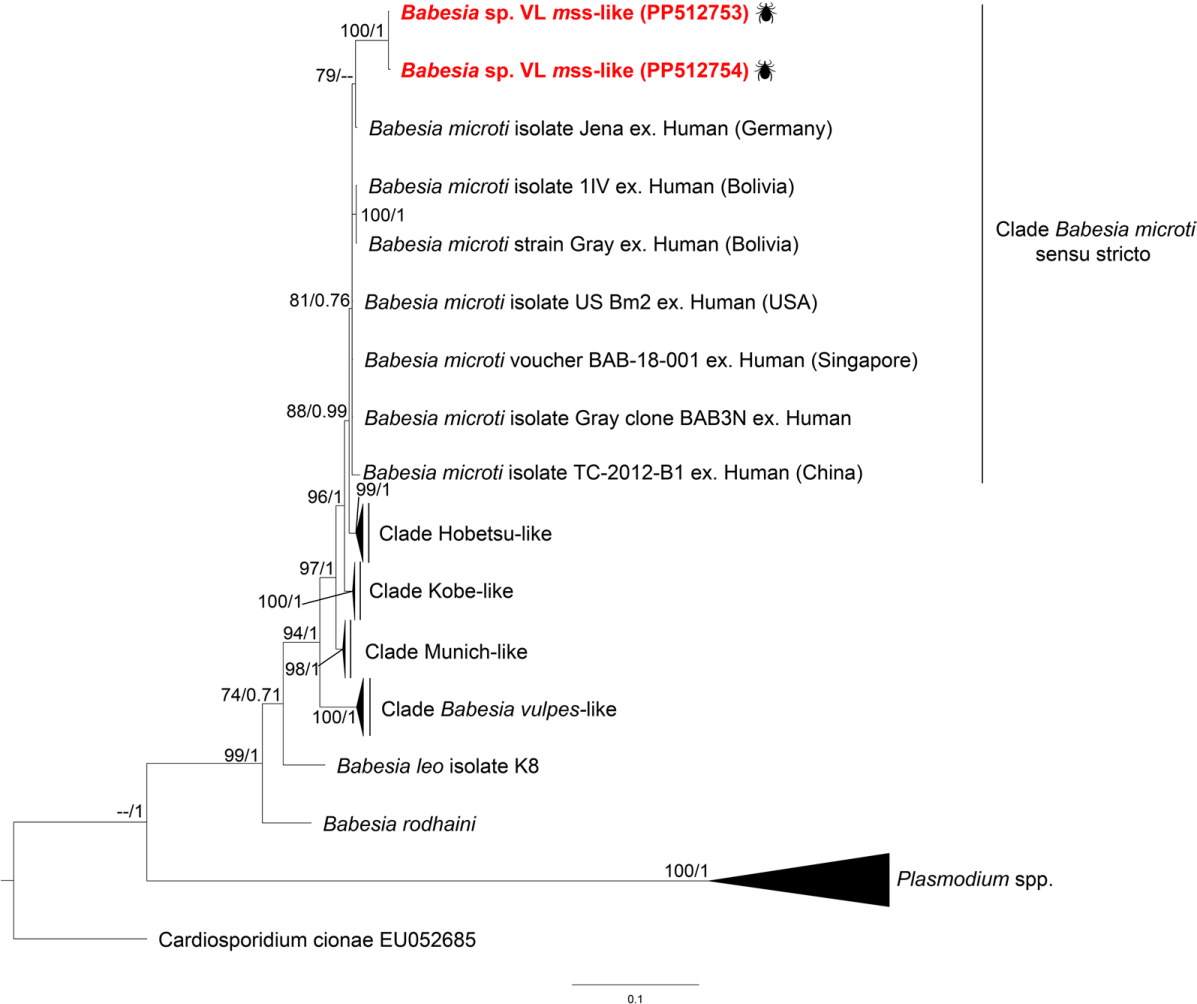
Maximum likelihood (ML) and Bayesian inference (BI) consensus trees of *Babesia*, highlighting the phylogenetic placement of *Babesia* sp. VL *m*s.s.-like (labeled in red font). Phylogenetic analyses were conducted, incorporating 43 sequences for the *18S* rRNA gene (aligned for 1722 bp). The best-fit evolutionary ML and BI models were determined for each gene dataset. For the ML, the model was TVM + F + G4, while the BI models included *M*_134_, *M*_200_,* M*_198_, *M*_85_,* M*_189_,* M*_203_. Only nodes with ultrafast bootstrap values > 70% for ML [64] and Bayesian posterior probabilities ≥ 0.71 for BI [67] are displayed above or below each branch. The scale bar represents the number of nucleotide substitutions per site. Details and GenBank accession numbers of sequences in collapse clades are provided in Additional File 5: Supplementary Table S5

**Fig. 4:**
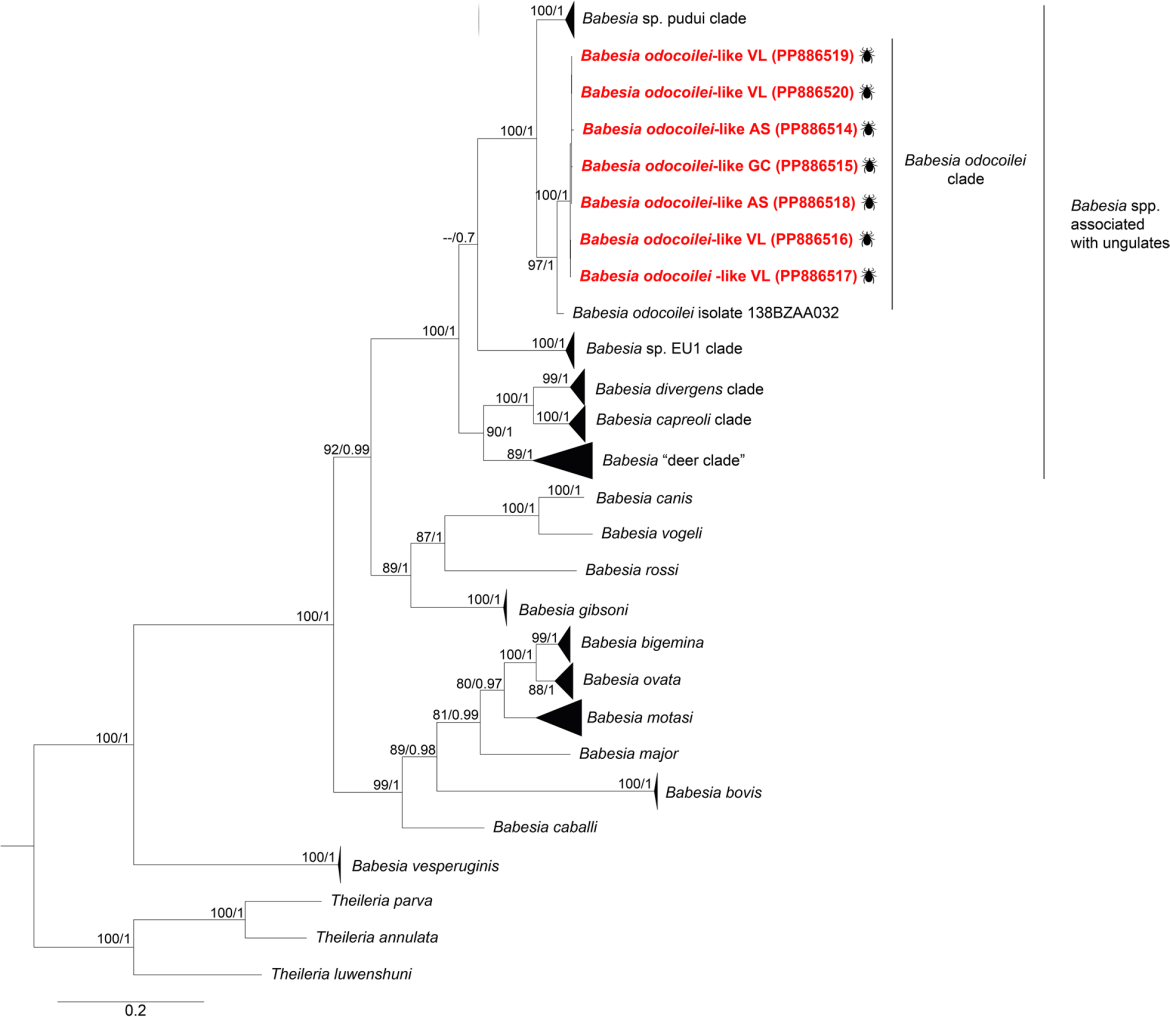
Maximum likelihood (ML) and Bayesian inference (BI) consensus trees of Piroplasmida, highlighting the phylogenetic placement of *Babesia odocoilei*-like (labeled in red font). Phylogenetic analyses were conducted incorporating 66 sequences for the *COI* gene (aligned for 1437 bp). The best-fit evolutionary ML and BI models were determined for each gene dataset. For the ML, the model was TN + F + G4 (position-1); TVM + F + G4 (position-2); TIM2 + F + G4 (position-3), while the BI models included *M*_40_, *M*_136_, *M*_125_, *M*_191_, *M*_138_ (position-1); *M*_138_, *M*_196_, *M*_68_, *M*_129_, *M*_154_ (position-2); and *M*_134_, *M*_200_, *M*_189_, *M*_198_, *M*_203_ (position-3). Only nodes with ultrafast bootstrap values > 70% for ML [64] and Bayesian posterior probabilities ≥ 0.71 for BI [67] are displayed above or below each branch. The scale bar represents the number of nucleotide substitutions per site. Details and GenBank accession numbers of sequences in collapse clades are provided in Additional File 5: Supplementary Table S5

Phylogenies inferred from both the *18S* rRNA and *COI* genes clustered our *B. odocoilei*-like genotypes, together with previously identified *B*. *odocoilei* sequences in Canada and the USA, forming the *B. odocoilei* clade (Figs. 2 and 4). However, the *18S* rRNA genotypes of *Babesia* sp. Valle del Lunarejo were placed within the *B. microti* s.s. clade (Fig. 3). Remarkably, *COI* gene amplification was unsuccessful for these samples.

## Discussion

This study provides the first genetic evidence of previously undescribed *Babesia* lineages in questing *I*. *fuscipes* ticks collected from various locations in Uruguay. A lineage represents an evolutionary line extending from a present-day species back to its ancestors, defined by their sequences [45, 46, 69]. Therefore, *Babesia* lineages identified in this study are considered as putatively undescribed species, with unique evolutionary trajectories shaped by genetic and ecological pressures that likely reflect specific adaptations of *Babesia* spp. to distinct host species and environments [12, 13, 17, 70].

The identification of *Babesia* lineages in *I*. *fuscipes* is consistent with its membership in the *I*. *ricinus* complex, which includes species such as *Ixodes scapularis* and *I*. *ricinus*, both well-recognized for harboring tick-borne infectious agents of the genera *Babesia*, *Borrelia*, and *Anaplasma* in the Northern Hemisphere [7, 12, 71]. To the best of our knowledge, this finding represents the first record of *B. odocoilei* and *B. microti* s.s. lineages in the *I*. *ricinus* complex from South America, marking a significant geographic expansion of lineages related to these groups beyond their previously known distributions. On the basis of their genetic and ecological distinctions, we propose the provisional names *Babesia odocoilei*-like and *Babesia* sp. Valle del Lunarejo *microti* s.s.-like (VL *m*s.s.-like) for these newly identified lineages, pending formal species descriptions. Prior genetic studies in Uruguay have documented *Ehrlichia* genotypes and *B. burgdorferi* sensu lato in *Ixodes auritulus* and *I. fuscipes* [35, 72], but this is the first study to associate *Babesia* spp. with *I. fuscipes*.

The unmasking of *B. odocoilei*-like and *Babesia* sp. VL *m*s.s.-like in Uruguay aligns with recent findings across the Neotropics. Over the past decade, research in this region has progressively uncovered *Babesia* lineages associated with *B*. *odocoilei* and the *B*. *microti*-like group in a variety of hosts and tick species. Lineages related to the former have been identified in *Ixodes* cf. *boliviensis* in Panama [73] and in *Ixodes stilesi* parasitizing pudu cervids (*Pudu puda*) in Chile [21]. As for the *B*. *microti*-like group, *B*. *microti* s.s. has been reported in Bolivian Chaco dwellers [74] and in a resident of Ecuador [75]. Other members of this group have been identified in a *Phyllotis darwini* rodent in Chile [22]. Most recently, another *B*. *microti*-like lineage, related to *Babesia vulpes*-like clade, was identified in an *Ixodes montoyanus* tick parasitizing an Andean bear (*Tremarctos ornatus*) in Ecuador [23].

The overall prevalence of *Babesia* DNA in *I. fuscipes* ticks (3.42%) exceeds the global average observed for ticks in the *I. ricinus* complex (0.4–1.9%) [76–78]. Interestingly, in Slovenia, the prevalence of *Babesia* in *I*. *ricinus* ticks collected from vegetation ranged from 7.4% to 9.6%, depending on the primers used [79]. The higher infection rate of *B. odocoilei*-like (2.68%) compared with *Babesia* sp. VL *m*s.s.-like (0.73%) points to a dominant presence of *B. odocoilei*-like in the surveyed ticks and localities. While *Babesia* species are commonly transmitted by *Ixodes* ticks [12, 13], the detection of *Babesia* DNA only suggest potential infection, and does not necessarily mean that the parasite can fully develop or persist in the host [13]. Additional testing would be required to determine this.

Higher prevalences of *B*. *odocoilei*-related lineages have been reported in other Neotropical *Ixodes* ticks, including 3.7% (1/27) in *I*. cf. *boliviensis* in Panama [73] and 20% (4/20) in *I*. *stilesis* in Chile [21]. It is worth noting that Santodomingo et al. [21] also identified the same *B*. *odocoilei*-related lineage in 10.9% (6/55) of pudues examined. Taken together, the findings across the Neotropics reinforce the potential role of *Ixodes* spp. as key vectors of *Babesia* spp. in diverse Neotropical environments and local enzootic scenarios. Furthermore, dissimilarities in prevalences, highlighting significant regional variability, may be attributed to ecological factors, such as climate, the availability and diversity of hosts, landscape settings, or methodological differences in *Babesia* detection protocols [79].

The high genetic similarities (Additional File 2: Supplementary Table S2) and phylogenetic relationships (Figs. 2 and 4) between *B. odocoilei*-like sequences and previously characterized *B. odocoilei* sequences from cervids and *Ixodes* ticks in North America [80, 81], along *B*. *odocoilei*-related lineages identified in pudues and *I*. *stilesi* ticks in Chile [21], indicate a shared evolutionary history of *B. odocoilei* and related lineages across the Americas. This evolutionary connection is likely driven by the strong association of *B*. *odocoilei* and related lineages with artiodactyl hosts, as reflected in the phylogenetic clustering of these lineages within the *Babesia*-associated ungulate clade [17]. In Uruguay, the gray brocket deer (*Subulo guazoubira*) is one of the most abundant deer species within the distribution range of *I. fuscipes*. Considering that this cervid is one of the main hosts of *I. fuscipes* [5, 6, 29], it would be necessary to investigate the possibility that the *B. odocoilei*-like identified in *I. fuscipes* could infect the gray brocket deer.

While the role of birds in the sylvatic cycles of *B*. *odocoilei* remains unclear, it has been found that *I*. *scapularis* ticks carried by migratory birds in Canada can harbor *B*. *odocoilei* DNA, suggesting that birds may act as spreaders of *B*. *odocoilei*-infected ticks [82]. Given that *I*. *fuscipes* also parasites birds during their immature stages, it is pertinent to assess the role of Neotropical birds in the propagation of *B*. *odocoilei*-related lineages. Importantly, the detection of *B. odocoilei*-like DNA in unfed larvae raises the possibility of transovarial transmission in *I. fuscipes*, a feature documented in both *B*. *odocoilei* and other *Babesia* s.s. lineages [12]. This hypothesis warrants further research to confirm the vertical transmission capabilities of the *B. odocoilei*-like in *I*. *fuscipes*.

Although *B*. *odocoilei* was initially considered non-pathogenic to cervids, infections have been associated with clinical signs under certain conditions, particularly in stressed or immunocompromised animals [70, 81, 83, 84]. Furthermore, *B*. *odocoilei* has recently been implicated in human babesiosis, highlighting its zoonotic potential [19]. Although no cases of *I. fuscipes* parasitizing humans have been reported in Uruguay, the high genetic similarity (Additional File 2: Supplementary Table S2, Additional File 4: Supplementary Table S4, and Figs. 2, 4) between the *B. odocoilei*-like and *B*. *odocoilei* raises the possibility that this lineage might also be capable of infecting humans. Further research is needed to evaluate the impact of *B. odocoilei*-like infection on native vertebrates in the region and its zoonotic potential.

However, sequences characterized from three *I. fuscipes* nymphs collected in Valle del Lunarejo showed high genetic similarity to *B. microti* sequences previously identified in humans, *Ixodes* ticks, and wild cricetid rodents in the Northern Hemisphere (Additional File 3: Supplementary Table S3). In addition, phylogenetic analysis further placed these sequences within the *B. microti* group (Fig. 3), specifically, within the *B*. *microti* s.s. clade. Members of this group lack the ability for transovarial transmission, which explains the detection of *B. microti* DNA only in nymphs. The presence of *Babesia* sp. VL *m*s.s.-like sequences in *I. fuscipes* suggests the involvement of local vertebrate reservoirs in its life cycle. This life cycle trait highlights the reliance of this parasite on competent vertebrate reservoirs at each tick stage.

In North America, *Peromyscus* rodents are well-known reservoirs, but in South America this role remains unclear. Findings in native cricetids from Chile support the idea that South American rodents may also serve as competent hosts [22]. In Uruguay, cricetid rodents are among the most common small mammals and are known hosts for immature *I. fuscipes* stages [29], making them plausible candidates as local reservoirs. Host competence studies involving these rodents would be essential to test their potential role in maintaining this parasite in the region. Although *I. fuscipes* also parasitizes birds during its larval and nymphal stages, *B. microti* has not been associated with avian hosts, suggesting a limited role for birds in its transmission cycle. Further investigation is required to evaluate whether other vertebrates might contribute to the maintenance or dissemination of this lineage in the region.

In our dataset, the prevalence of *Babesia* sp. VL *m*s.s.-like was 0.73%, lower than that of *B. odocoilei*-like (2.68%), suggesting a less widespread or less established presence of this lineage in the surveyed areas. Despite its lower prevalence, the presence of *B. microti*-like lineages in diverse vertebrate and tick hosts across South America reinforces the ecological plasticity and expanding distribution of this group in the region [22, 23].

It is well-documented that *B*. *microti* s.s. lineages are the primary etiological agent of human babesiosis in the northern latitudes worldwide. In the Americas, human cases have been reported in Canada, Mexico, and Ecuador and asymptomatic cases have been documented in Bolivia and Brazil [74, 75, 85]. However, its potential occurrence in Uruguay had remained unclear until now. Although endemic to the Northern Hemisphere, its detection in Uruguay and other South American countries [74, 75, 85] underscores the importance of continued surveillance to evaluate its epidemiological significance. Finally, as with *B. odocoilei*-like, detection of *B. microti* DNA alone does not confirm vector competence of *I. fuscipes*, and experimental transmission studies, as well as host competence assays in local rodent species, are essential to fully elucidate its epidemiological role in Uruguay.

## Conclusions

This study documents *B*. *odocoilei* and *B*. *microti* s.s.-related lineages identified in questing *I*. *fuscipes* ticks in Uruguay, offering insights into the diversity of piroplasms in the region. The identification of *B. odocoilei*-like and *Babesia* sp. VL *m*s.s.-like add to the growing body of evidence about the occurrence of *Babesia* lineages associated with *Babesia* zoonotic lineages in South American wildlife. Within this context, tick-borne disease surveillance, as exemplified by this study, is essential for establishing early warning systems to detect and mitigate the emergence of diseases associated with these arthropods. Future research should focus on identifying potential vertebrate hosts, elucidating the role of *I*. *fuscipes* in vector-borne transmission and assessing the pathogenicity and zoonotic potential of these lineages. Expanding surveillance to include other tick species and vertebrate hosts will provide critical insights into the dynamics of *Babesia* infections in the region.

## Supplementary Information

Additional File 1: Supplementary Table S1. BLASTn results of partial 16S rRNA sequences of *Ixodes fuscipes* collected from Uruguay and other ticks of *I. ricinus* complex in South Cone of South America.

Additional File 2: Supplementary Table S2. BLASTn results of *18S* rRNA sequences of *Babesia odocoilei*-like obtained in this study.

Additional File 3: Supplementary Table S3. BLASTn results of *18S* rRNA sequences of *Babesia* sp. VL *m*ss-like obtained in this study.

Additional File 4: Supplementary Table S4. BLASTn results of *COI* sequences of *Babesia odocoilei*-like obtained in this study.

Additional File 5: Supplementary Table S5. Details and GenBank accession numbers of sequences used in collapse clades.

## Data Availability

The data supporting the findings of the study are provided within the manuscript or supplementary information files.

## Abbreviations

*18S* rRNA: *18**S* ribosomal RNA
s.s.: Sensu stricto
DNA: Deoxyribonucleic acid
*COI*: Cytochrome c oxidase subunit 1
*cytb*: Cytochrome *b*
AS: Arroyo Sepulturas
VL: Valle del Lunarejo
PA: Puntas de Arapey
GC: Gruta de los Cuervos
LN: Laguna Negra
m: Meter
16S rRNA: 16S ribosomal rRNA
μL: Microliter
mM: Millimolar
Tris–Cl: Tris hydrochloride
EDTA: Ethylenediaminetetraacetic acid
PCR: Polymerase chain reaction
bp: Base pairs
UV: Ultraviolet
BLASTn: Basic local alignment search tool
MAFFT: Multiple sequence alignment program
BMGE: Block Mapping and Gathering with Entropy
ML: Maximum likelihood
BI: Bayesian inference
UFBoot: Ultrafast bootstrap
MCMC: Markov Chain Monte Carlo
ESS: Effective sample size
BPP: Bayesian posterior probabilities
mt: Mitochondrial
USA: United States of America
*Babesia * sp. Valle del Lunarejo *microti * s.s.-like: *Babesia* sp. VL *m*s.s.-like
