# Supplementary material for: A survey for piroplasmids in questing Ixodes fuscipes ticks reveals undescribed Babesia lineages in Uruguay
Source: Parasit Vectors. 2025 Jun 18;18:225. doi: 10.1186/s13071-025-06866-0 (PMC12175381; doi:10.1186/s13071-025-06866-0)
Supplement: Supplementary file 1 — Additional File 1: Supplementary Table S1. BLASTn results of partial 16S rRNA sequences of Ixodes fuscipes collected from Uruguay and other ticks of I. ricinus complex in South Cone of South America. [file 13071_2025_6866_MOESM1_ESM.docx]

**Additional file 1: Table S1**. BLASTn results of partial 16S rRNA sequences of *Ixodes fuscipes* collected from Uruguay and other ticks of *I. ricinus* complex in South Cone of South America.

| **Sample ID of *Ixodes fuscipes* of this study (location, stage, GenBank acc. number, sequence size)** | **Query coverage (%)** | **Identity (%)** | **Gaps** | **E-value** | **Species of *I. ricinus complex,* sequences in GenBank*: acc. number, country, sequence size** |
| --- | --- | --- | --- | --- | --- |
| S31IpH1 (LN, female, PQ868241, 432 bp) | 100% | 99.77% | 1/432 | 0.0 | *I. fuscipes,* KU894400, Brazil, 439 bp.  *I. fuscipes,* KU894395, Brazil, 451 bp.  *I. fuscipes,* KU894398, Brazil, 451 bp. |
|  | 95% | 99.27% | 1/411 | 0.0 | *I. fuscipes,* JX082322, Uruguay, 410 bp. |
|  | 87% | 99.20% | 1/377 | 0.0 | *I. fuscipes,* KJ650033, Uruguay, 376 bp. |
|  | 98% | 94.33% | 3/423 | 1e-180 | *I. chacoensis,* MT604112, Argentina, 420 bp |
|  | 99% | 93.24% | 5/429 | 5e-175 | *I. pararicinus,* KY610209, Argentina, 425 bp |
|  | 99% | 93.22% | 5/428 | 2e-174 | *I. pararicinus,* KY610208, Argentina,424 bp |
|  | 99% | 93.02% | 5/430 | 7e-174 | *I. pararicinus,* AF549855, Argentina, 479pb |
| S32IpM21 (PA, male, PQ868242, 432 bp) | 100% | 99.77% | 1/432 | 0.0 | *I. fuscipes,* KU894401, Brazil, 440 bp.  *I. fuscipes,* KU894396, Brazil, 451 bp.  *I. fuscipes,* KU894392, Brazil, 451 bp. |
|  | 95% | 99.03% | 1/411 | 0.0 | *I. fuscipes,* JX082322, Uruguay, 410bp. |
|  | 98% | 94.09% | 3/423 | 7e-179 | *I. chacoensis,* MT604112, Argentina, 420 bp |
|  | 99% | 93.47% | 5/429 | 1e-176 | *I. pararicinus,* KY610209, Argentina, 425 bp |
|  | 99% | 93.46% | 5/428 | 1e-176 | *I. pararicinus,* KY610208, Argentina,424 bp |
|  | 99% | 93.26% | 5/430 | 1e-175 | *I. pararicinus,* AF549855, Argentina, 479pb |
| S34IpH1 (AS, female, PQ868243, 431 bp) | 95% | 100% | 0/410  0/376 | 0.0 | *I. fuscipes,* JX082322, Uruguay, 410 bp.  *I. fuscipes,* KJ650033, Uruguay, 376 bp. |
|  | 100% | 99.54% | 1/432  0/431 | 0.0 | *I. fuscipes,* KU894397, Brazil,447 bp.  *I. fuscipes,* KU894400, Brazil, 439 bp. |
|  | 92% | 99.24% | 2/396 | 0.0 | *I. fuscipes,* KX231831, Brazil, 394 bp. |
|  | 99% | 93.84% | 2/422 | 3e-177 | *I. chacoensis,* MT604112, Argentina, 420 bp |
|  | 99% | 93.71% | 6/429 | 2e-178 | *I. pararicinus,* KY610209, Argentina, 425 bp |
|  | 99% | 93.69% | 6/428 | 9e-178 | *I. pararicinus,* KY610208, Argentina,424 bp |
|  | 99% | 93.49% | 6/430 | 3e-177 | *I. pararicinus,* AF549855, Argentina, 479pb |
| S39IpM1 (GC, male,  PQ868244, 403 bp) | 100% | 100% | 0/403 | 0.0 | *I. fuscipes,* KU894400, Brazil, 439 bp.  *I. fuscipes,* KU894395, Brazil, 451 bp.  *I. fuscipes,* KU894398, Brazil, 451 bp. |
|  | 97% | 99.49% | 0/390 | 0.0 | *I. fuscipes,* JX082322, Uruguay, 410 bp. |
|  | 93% | 99.47% | 0/376 | 0.0 | *I. fuscipes,* KJ650033, Uruguay, 376 bp. |
|  | 100 | 94.03% | 2/402 | 2e-169 | *I. chacoensis* MT604112, Argentina, 420 bp  *I. chacoensis,* MT604113, Argentina, 420 bp  *I. chacoensis,* MT604111, Argentina, 420 bp  *I. chacoensis,* MT604114, Argentina, 420 bp |
|  | 100% | 93.32% | 4/404 | 5e-165 | *I. pararicinus,* AF549855, Argentina, 479pb |
|  | 100% | 93.28% | 4/402 | 6e-164 | *I. pararicinus,* KY610209, Argentina, 425 bp |
|  | 99% | 93.27% | 4/401 | 2e-163 | *I. pararicinus,* KY610208, Argentina,424 bp |
| S39IpN37 (VL, nymph, PQ868245, 431 bp) | 95% | 100% | 0/410 | 0.0 | *I. fuscipes,* JX082322, Uruguay, 410 bp. |
|  | 87% | 100% | 0/376 | 0.0 | *I. fuscipes,* KJ650033, Uruguay, 376 bp. |
|  | 100% | 99.54% | 1/432  0/431  0/431 | 0.0 | *I. fuscipes,* KU894397, Brazil, 447 bp.  *I. fuscipes,* KU894400, Brazil, 439 bp.  *I. fuscipes,* KU894395, Brazil, 451 bp. |
|  | 98% | 93.84% | 2/422 |  | *I. chacoensis,* MT604113, Argentina, 420 bp  *I. chacoensis,* MT604111, Argentina, 420 bp  *I. chacoensis,* MT604114, Argentina, 420 bp |
|  | 99% | 93.71% | 6/429 | 2e-178 | *I. pararicinus,* KY610209, Argentina, 425 bp |
|  | 99% | 93.69% | 6/428 | 9e-178 | *I. pararicinus,* KY610208, Argentina,424 bp |
|  | 99% | 93.49% | 6/430 | 3e-177 | *I. pararicinus,* AF549855, Argentina, 479pb |
| S44IpL13 (VL, larva,  PQ868246, 431 bp) | 95% | 99.51% | 0/410 | 0.0 | *I. fuscipes,* JX082322, Uruguay, 410 bp. |
|  | 87% | 99.47% | 0/376 | 0.0 | *I. fuscipes,* KJ650033, Uruguay, 376 bp. |
|  | 100% | 99.07% | 1/432  0/431  0/431 | 0.0 | *I. fuscipes,* KU894397, Brazil, 447 bp.  *I. fuscipes,* KU894400, Brazil, 439 bp.  *I. fuscipes,* KU894395, Brazil, 451 bp. |
|  | 99% | 93.47% | 6/429 | 1e-176 | *I. pararicinus,* KY610209, Argentina, 425 bp |
|  | 99% | 93.46% | 6/428 | 1e-176 | *I. pararicinus,* KY610208, Argentina,424 bp |
|  | 98% | 93.38% | 4/423 | 7e-174 | *I. chacoensis,* MT604112, Argentina, 420 bp  *I. chacoensis,* MT604113, Argentina, 420 bp  *I. chacoensis,* MT604111, Argentina, 420 bp  *I. chacoensis,* MT604114, Argentina, 420 bp |
|  | 99% | 93.26% | 6/430 | 1e-175 | *I. pararicinus,* AF549855, Argentina, 479pb |

* Some sequences are determined in GenBank as *Ixodes pararicinus* and *Ixodes aragaoi*, but currently in Uruguay and Brazil, they correspond to *Ixodes fuscipes*, and *Ixodes* cf *affinis* in Argentina correspond to *Ixodes chacoensis*.
