# Supplementary material for: A survey for piroplasmids in questing Ixodes fuscipes ticks reveals undescribed Babesia lineages in Uruguay
Source: Parasit Vectors. 2025 Jun 18;18:225. doi: 10.1186/s13071-025-06866-0 (PMC12175381; doi:10.1186/s13071-025-06866-0)
Supplement: Supplementary file 2 — Additional File 2: Supplementary Table S2. BLASTn results of 18S rRNA sequences of Babesia odocoilei-like obtained in this study. [file 13071_2025_6866_MOESM2_ESM.docx]

**Additional file 2: Table S2.** BLASTn results of *18S* rRNA sequences of *Babesia odocoilei*-like obtained in this study.

| **Sample ID (location, stage, GenBank acc. number, sequence size)** | **Query coverage %** | **Identity %** | **Gaps** | **E-value** | **Species or genotypes (GenBank acc. Number, Country, sequence size)** |
| --- | --- | --- | --- | --- | --- |
| S21IpN3 (VL, nymph, PP512757, 1425 bp) | 100% | 99.51% | 1/1426  1/1426  1/1426  1/1426  1/1426  1/1426  1/1426  0/1425 | 0.0 | *Babesia odocoilei* (U16369, USA, 1723 bp)  *Babesia odocoilei* (AY661507, USA, 1605 bp)  *Babesia odocoilei* (MF357057, Canada, 1611 bp)  *Babesia odocoilei* (AY661503, USA, 1608 bp)  *Babesia odocoilei* (KC460321, Canada, 1607 bp)  *Babesia odocoilei* (AY046577, USA, 1727 bp)  *Babesia odocoilei* (MF357056, Canada, 1618 bp)  *Babesia* sp. pudui (ON994405, Chile, 1428 bp) |
|  | 99% | 99.51% | 0/1416 |  | *Babesia* sp. pudui (ON994403, Chile, 1416 bp) |
|  | 96% | 99.49% | 0/1373 |  | *Babesia* sp. pudui (ON994402, Chile, 1373 bp) |
|  | 100% | 99.44% | 1/1426  1/1426  0/1423 |  | *Babesia odocoilei* (AY661509, USA, 1658 bp)  *Babesia odocoilei* (AY661502, USA, 1605 bp)  *Babesia* sp. pudui (ON994406, Chile, 1426 bp) |
| S22IpN4 (VL, nymph, PV061833, 488 bp) | 100% | 99.39% | 0/488  0/488  0/488  0/488  0/488  0/488  0/488  0/488  1/488  1/488  1/488 | 0.0 | *Babesia* sp. pudui (ON994402, Chile, 1373 bp)  *Babesia* sp. pudui (ON994405, Chile,1428 bp)  *Babesia* sp. pudui (ON994403, Chile, 1416 bp) |
|  | 100% | 99.18% |  |  | *Babesia* sp. pudui (ON994407, Chile, 1389 bp)  *Babesia* sp. pudui (ON994409, Chile, 1360 bp)  *Babesia* sp. pudui (ON994406, Chile, 1426 bp)  *Babesia* sp. pudui (ON994401, Chile, 1379 bp)  *Babesia* sp. pudui (ON994408, Chile, 1331 bp)  *Babesia odocoilei* (U16369, USA,1723 bp)  *Babesia odocoilei* (AY661507, USA ,1605 bp)  *Babesia odocoilei* (MF357057, Canada, 1611 bp) |
| S32IpL12 (AS, larva, PV061834, 486 bp) | 100% | 99.38% | 0/486 | 0.0 | *Babesia* sp. pudui (ON994402, Chile, 1373 bp)  *Babesia* sp. pudui (ON994405, Chile, 1428 bp)  *Babesia* sp. pudui (ON994403, Chile, 1416 pb) |
|  | 100% | 99.18% | 0/486 |  | *Babesia* sp. pudui (ON994407, Chile, 1389 bp)  *Babesia* sp. pudui (ON994409, Chile, 1360 bp)  *Babesia* sp. pudui (ON994406, Chile, 1426 bp)  *Babesia* sp. pudui (ON994401, Chile, 1379 bp)  *Babesia* sp. pudui (ON994408, Chile, 1331 bp) |
|  | 100% | 98.97% | 0/486 |  | *Babesia odocoilei* (U16369, USA, 1723 bp)  *Babesia odocoilei* (AY661507, USA, 1605 bp)  *Babesia odocoilei* (MF357057, Canada, 1611 bp) |
| S32IpN18 (AS, nymph, PP512756, 1414 bp) | 100% | 99.51% | 1/1415 | 0.0 | *Babesia odocoilei* (U16369, USA, 1723 pb)  *Babesia odocoilei* (AY661507, USA, 1605 bp)  *Babesia odocoilei* (MF357057, Canada, 1611 bp)  *Babesia odocoilei* (AY661503, USA, 1608 pb)  *Babesia odocoilei* (KC460321, Canada, 1607 bp)  *Babesia odocoilei* (AY046577, USA, 1727 bp)  *Babesia odocoilei* (MF357056, Canada, 1618 bp) |
|  | 100% | 99.50% | 0/1414  0/1408 |  | *Babesia* sp. pudui (ON994405, Chile, 1428 bp)  *Babesia* sp. pudui (ON994403, Chile, 1416 pb) |
|  | 97% | 99.49% | 0/1373 |  | *Babesia* sp. pudui (ON994402, Chile, 1373 bp) |
|  | 100% | 99.43% | 1/1415  1/1415  0/1414 |  | *Babesia odocoilei* (AY661509, USA, 1658 bp)  *Babesia odocoilei* (AY661502, USA, 1605 bp)  *Babesia* sp. pudui (ON994406, Chile, 1426 bp) |
| S36IpN5 (VL, nymph, PV061835, 960 bp) | 100% | 99.58% | 0/960  0/960  0/960  0/960  0/960  0/960  0/960  0/960  0/960  0/960  0/960 | 0.0 | *Babesia odocoilei* (AY661510, USA, 1658 bp)  *Babesia odocoilei* (MH366540, Canada , 1093 bp)  *Babesia odocoilei* (U16369, USA, 1723 pb)  *Babesia odocoilei* (AY661507, USA, 1605 bp)  *Babesia odocoilei* (MF357057, Canada, 1611 bp)  *Babesia odocoilei* (AY144689, USA, 1225 bp)  *Babesia odocoilei* (AY661503, USA, 1608 bp)  *Babesia odocoilei* (MH366302, Canada, 1290 bp)  *Babesia odocoilei* (KC460321, Canada, 1607 bp)  *Babesia odocoilei* (AY046577, USA, 1727 bp)  *Babesia odocoilei* (MF357056, Canada, 1618 bp) |
|  | 100% | 99.48% | 0/960  0/960  0/960  0/960  0/960  0/959 |  | *Babesia odocoilei* (AY661509, USA, 1658 bp)  *Babesia odocoilei* (AY661502, USA, 1605 bp)  *Babesia odocoilei* (OR268712, Scotland, 1013 bp)  *Babesia* sp. pudui (ON994405, Chile, 1428 bp)  *Babesia odocoilei* (MH899097, Canada, 1093 bp)  *Babesia* sp. pudui (ON994403, Chile, 1416 pb) |
|  | 99% | 99.47% | 0/951 |  | *Babesia* sp. pudui (ON994402, Chile, 1373 bp) |
|  | 95% | 99.45% | 0/913 |  | *Babesia* sp. pudui (ON994404, Chile, 1149 bp) |
| S36IpN6 (VL, nymph, PV098357, 548 bp) | 100% | 99.45% | 0/548  0/548  0/548 | 0.0 | *Babesia* sp. pudui (ON994402, Chile, 1373 bp)  *Babesia* sp. pudui (ON994403, Chile, 1416 pb)  *Babesia* sp. pudui (ON994405, Chile, 1428 bp) |
|  | 100% | 99.27% | 0/548  0/548  0/548  0/548  0/548 |  | *Babesia sp. pudui* (ON994408, Chile, 1331 bp)  *Babesia* sp. pudui (ON994407, Chile, 1389 bp)  *Babesia* sp. pudui (ON994401, Chile, 1379 bp)  *Babesia* sp. pudui (ON994409, Chile, 1360 bp)  *Babesia* sp. pudui (ON994406, Chile, 1426 bp) |
|  | 100% | 99.09% | 0/548 |  | *Babesia* sp. pudui (ON994400, Chile, 1419 bp) |
|  | 100% | 98.91% | 1/549  1/549  1/549  1/549  1/549  1/549  1/549  1/549  1/549 |  | *Babesia odocoilei* (MF357056, Canada, 1618 bp)  *Babesia odocoilei* (AY661509, USA, 1658 bp)  *Babesia odocoilei* (U16369, USA, 1723 bp)  *Babesia odocoilei* (AY046577, USA, 1727 bp)  *Babesia odocoilei* (AY661503, USA, 1608 bp)  *Babesia odocoilei* (MF357057, Canada, 1611 bp)  *Babesia odocoilei* (AY661507, USA, 1605 bp)  *Babesia odocoilei* (AY661502, USA, 1605 bp)  *Babesia odocoilei* (KC460321, Canada, 1607 bp) |
| S39IpN17 (GC, nymph, PP512755, 1339 bp) | 100% | 99.48% | 1/1340  1/1340  1/1340  1/1340  1/1340  1/1340  1/1340  0/1339  0/1339  0/1339 | 0.0 | *Babesia odocoilei* (U16369, USA, 1723 pb)  *Babesia odocoilei* (AY661507, USA, 1605 bp)  *Babesia odocoilei* (MF357057, Canada, 1611 bp)  *Babesia odocoilei* (AY661503, USA, 1608 bp)  *Babesia odocoilei* (KC460321, Canada, 1607 bp)  *Babesia odocoilei* (AY046577, USA, 1727 bp)  *Babesia odocoilei* (MF357056, Canada, 1618 bp)  *Babesia* sp. pudui (ON994402, Chile, 1373 bp)  *Babesia* sp. pudui (ON994405, Chile, 1428 bp)  *Babesia* sp. pudui (ON994403, Chile, 1416 pb) |
|  | 100% | 99.40% | 1/1340  1/1340  0/1339  0/1339  0/1339  0/1333  0/1331 |  | *Babesia odocoilei* (AY661509, USA, 1658 bp)  *Babesia odocoilei* (AY661502, USA, 1605 bp)  *Babesia* sp. pudui (ON994407, Chile, 1389 bp)  *Babesia* sp. pudui (ON994406, Chile, 1426 bp)  *Babesia* sp. pudui (ON994401, Chile, 1379 bp)  *Babesia* sp. pudui (ON994409, Chile, 1360 bp)  *Babesia* sp. pudui (ON994408, Chile, 1331 bp) |
|  | 100% | 99.33 | 1/1340  0/1339 |  | *Babesia odocoilei* (AY661510, USA, 1658 bp)  *Babesia* sp. pudui (ON994400, Chile, 1419 bp) |
| S40IpN5 (VL, nymph, PV061836, 551 bp) | 98% | 99.44% | 0/539  0/539  0/539 | 0.0 | *Babesia* sp. pudui (ON994402, Chile, 1373 bp)  *Babesia* sp. pudui (ON994405, Chile, 1428 bp)  *Babesia* sp. pudui (ON994403, Chile, 1416 pb) |
|  | 98% | 99.26% | 0/539  0/539  0/539  0/539  0/539 |  | *Babesia* sp. pudui (ON994407, Chile, 1389 bp)  *Babesia* sp. pudui (ON994409, Chile, 1360 bp)  *Babesia* sp. pudui (ON994406, Chile, 1426 bp)  *Babesia* sp. pudui (ON994401, Chile, 1379 bp)  *Babesia* sp. pudui (ON994408, Chile, 1331 bp) |
|  | 98% | 99.07% | 0/539 |  | *Babesia* sp. pudui (ON994400, Chile, 1419 bp) |
|  | 98% | 98.89% | 1/540  1/540  1/540  1/540  1/540  1/540  1/540  1/540  1/540  1/540 |  | *Babesia odocoilei* (U16369, USA, 1723 pb)  *Babesia odocoilei* (AY661507, USA, 1605 bp)  *Babesia odocoilei* (MF357057, Canada, 1611 bp)  *Babesia odocoilei* (AY661503, USA, 1608 bp)  *Babesia odocoilei* (AY661509, USA, 1658 bp)  *Babesia odocoilei* (AY661502, USA, 1605 bp)  *Babesia odocoilei* (KC460321, Canada, 1607 bp)  *Babesia odocoilei* (AF158711, USA, 1671 bp)  *Babesia odocoilei* (AY046577, USA, 1727 bp)  *Babesia odocoilei* (MF357056, Canada, 1618 bp) |
| S40IpN9 (GC, nymph, PV061837, 557 bp) | 100% | 99.28% | 0/557  0/557  0/557 | 0.0 | *Babesia* sp. pudui (ON994402, Chile, 1373 bp)  *Babesia* sp. pudui (ON994405, Chile, 1428 bp)  *Babesia* sp. pudui (ON994403, Chile, 1416 pb) |
|  | 100% | 99.10% | 0/557  0/557  0/557 |  | *Babesia* sp. pudui (ON994407, Chile, 1389 bp)  *Babesia* sp. pudui (ON994406, Chile, 1426 bp)  *Babesia* sp. pudui (ON994401, Chile, 1379 bp) |
|  | 99% | 99.09% | 0/551  0/551 |  | *Babesia* sp. pudui (ON994409, Chile, 1360 bp)  *Babesia* sp. pudui (ON994408, Chile, 1331 bp) |
|  | 100% | 98.75% | 1/558  1/558  1/558  1/558  1/558  1/558  1/558  1/558  1/558  1/558 |  | *Babesia odocoilei* (U16369, USA, 1723 pb)  *Babesia odocoilei* (AY661507, USA, 1605 bp)  *Babesia odocoilei* (MF357057, Canada, 1611 bp)  *Babesia odocoilei* (AY661503, USA, 1608 bp)  *Babesia odocoilei* (AY661509, USA, 1658 bp)  *Babesia odocoilei* (AY661502, USA, 1605 bp)  *Babesia odocoilei* (KC460321, Canada, 1607 bp)  *Babesia odocoilei* (AF158711, USA, 1671 bp)  *Babesia odocoilei* (AY046577, USA, 1727 bp)  *Babesia odocoilei* (MF357056, Canada, 1618 bp |
| S44IpL20 (VL, larva, PV061838, 558 bp) | 100 | 99.64 | 0/558  0/558  0/558 | 0.0 | *Babesia* sp. pudui (ON994402, Chile, 1373 bp)  *Babesia* sp. pudui (ON994405, Chile, 1428 bp)  *Babesia* sp. pudui (ON994403, Chile, 1416 pb) |
|  | 100 | 99.46 | 0/558  0/558  0/558 |  | *Babesia* sp. pudui (ON994407, Chile, 1389 bp)  *Babesia* sp. pudui (ON994406, Chile, 1426 bp)  *Babesia* sp. pudui (ON994401, Chile, 1379 bp) |
|  | 99 | 99.46 | 0/552  0/552 |  | *Babesia* sp. pudui (ON994409, Chile, 1360 bp)  *Babesia* sp. pudui (ON994408, Chile, 1331 bp) |
|  | 100 | 98.75 | 1/559  1/559  1/559  1/559  1/559  1/559  1/559  1/559  1/559  1/559 |  | *Babesia odocoilei* (U16369, USA, 1723 pb)  *Babesia odocoilei* (AY661507, USA, 1605 bp)  *Babesia odocoilei* (MF357057, Canada, 1611 bp)  *Babesia odocoilei* (AY661503, USA, 1608 bp)  *Babesia odocoilei* (AY661509, USA, 1658 bp)  *Babesia odocoilei* (AY661502, USA, 1605 bp)  *Babesia odocoilei* (KC460321, Canada, 1607 bp)  *Babesia odocoilei* (AF158711, USA, 1671 bp)  *Babesia odocoilei* (AY046577, USA, 1727 bp)  *Babesia odocoilei* (MF357056, Canada, 1618 bp |
| S44IpN66 (AS, nymph, PV061839, 533 bp) | 100% | 99.44% | 0/533  0/533  0/533 | 0.0 | *Babesia* sp. pudui (ON994402, Chile, 1373 bp)  *Babesia* sp. pudui (ON994405, Chile, 1428 bp)  *Babesia* sp. pudui (ON994403, Chile, 1416 pb) |
|  | 100% | 99.25% | 0/533  0/533  0/533  0/533  0/533 |  | *Babesia* sp. pudui (ON994407, Chile, 1389 bp)  *Babesia* sp. pudui (ON994409, Chile, 1360 bp)  *Babesia* sp. pudui (ON994406, Chile, 1426 bp)  *Babesia* sp. pudui (ON994401, Chile, 1379 bp)  *Babesia* sp. pudui (ON994408, Chile, 1331 bp) |
|  | 100% | 99.06% | 0/533 |  | *Babesia* sp. pudui (ON994400, Chile, 1419 bp) |
|  | 100% | 98.88% | 1/534  1/534  1/534  1/534  1/534  1/534  1/534  1/534  1/534  1/534 |  | *Babesia odocoilei* (U16369, USA, 1723 pb)  *Babesia odocoilei* (AY661507, USA, 1605 bp)  *Babesia odocoilei* (MF357057, Canada, 1611 bp)  *Babesia odocoilei* (AY661503, USA, 1608 bp)  *Babesia odocoilei* (AY661509, USA, 1658 bp)  *Babesia odocoilei* (AY661502, USA, 1605 bp)  *Babesia odocoilei* (KC460321, Canada, 1607 bp)  *Babesia odocoilei* (AF158711, USA, 1671 bp)  *Babesia odocoilei* (AY046577, USA, 1727 bp)  *Babesia odocoilei* (MF357056, Canada, 1618 bp) |
