# Supplementary material for: A survey for piroplasmids in questing Ixodes fuscipes ticks reveals undescribed Babesia lineages in Uruguay
Source: Parasit Vectors. 2025 Jun 18;18:225. doi: 10.1186/s13071-025-06866-0 (PMC12175381; doi:10.1186/s13071-025-06866-0)
Supplement: Supplementary file 3 — Additional File 3: Supplementary Table S3. BLASTn results of 18S rRNA sequences of Babesia sp. VL mss-like obtained in this study. [file 13071_2025_6866_MOESM3_ESM.docx]

**Additional file 3: Table S3.** BLASTn results of *18S* rRNA sequences of *Babesia* sp. VL *m*ss-like obtained in this study.

| **Sample ID (location, stage, GenBank acc. Number, sequence size)** | **Query coverage %** | **Identity %** | **Gaps** | **E-value** | **Species or genotypes (GenBank acc. number, country, sequence size)** |
| --- | --- | --- | --- | --- | --- |
| S35IpN6 (VL, nymph, PP512753, 1477 bp) | 100% | 98.44% | 3/1479  3/1479 | 0.0 | *Babesia microti* (EF413181, Germany, 1687 bp)  *Babesia microti* (AB083375, China, 1665 bp) |
|  | 100% | 98.38% | 3/1479  3/1479  3/1479  3/1479  3/1479  3/1479  3/1479  3/1479  3/1479  3/1479  3/1479  3/1477  3/1477 |  | *Babesia microti* (AB085191, Germany, 1721 bp)  *Babesia microti* (LC314655, USA, 1612 bp)  *Babesia microti* (MT423327, China, 1702 bp)  *Babesia microti* (AF231348, USA, 1705 bp)  *Babesia microti* (LC127369, Japan, 1577 bp)  *Babesia microti* (MK609547, USA, 2573 bp)  *Babesia microti* (LC005768, Mongolia, 1667 bp)  *Babesia microti* (AB190435, Japan, 2525 bp)  *Babesia microti* (LC005771, Mongolia, 1667 bp)  *Babesia microti* (LC005752, Mongolia, 1667 bp)  *Babesia microti* (AB190459, Japan, 2536 bp)  *Babesia microti* (LC127372, Japan, 1573 pb)  *Babesia microti* (LC127371, Japan, 1577 bp) |
| S35IpN9 (VL, nymph, PP512754, 1474 bp) | 100% | 98.37% | 1/1475  1/1475 | 0.0 | *Babesia microti* (EF413181, Germany, 1687 bp)  *Babesia microti* (AB083375, China, 1665 bp) |
|  | 100% | 98.31% | 1/1475  1/1475  1/1475  1/1475  1/1475  1/1475  1/1475  1/1475  1/1475  1/1475  1/1475  1/1475  1/1475 |  | *Babesia microti (AB085191,* Germany, 1721 bp)  *Babesia microti (LC314655,* USA, 1612 bp)  *Babesia microti (MT423327,* China, 1702 bp)  *Babesia microti* (AF231348, USA, 1705 bp)  *Babesia microti* (LC127369, Japan, 1577 bp)  *Babesia microti* (MK609547, USA, 2573 bp)  *Babesia microti* (LC005768, Mongolia, 1667 bp)  *Babesia microti* (LC127372, Japan, 1573 pb)  *Babesia microti* (LC127371, Japan, 1577 bp)  *Babesia microti* (AB190435, Japan, 2525 bp)  *Babesia microti* (LC005771, Mongolia, 1667 bp)  *Babesia microti* (LC005752, Mongolia, 1667 bp)  *Babesia microti* (AB190459, Japan, 2536 bp) |
| S40IpN3 (VL, nymph, PV053416, 523 bp) | 100% | 97.14% | 3/525  3/525  3/525 | 0.0 | *Babesia microti* (EF413181, Germany, 1687 bp)  *Babesia microti* (AB083375, China, 1665 bp)  *Babesia microti* (OM066130, Turkey, 966 bp) |
|  | 100% | 96.95% | 3/525  3/525  3/525  3/525  3/525  3/525  3/525  3/525  3/525  3/525  3/525  3/525 |  | *Babesia microti* (JX417370, Australia, 805 bp)  *Babesia microti* (AB085191, Germany, 1721 bp)  *Babesia microti* (LC314655, USA, 1612 bp)  *Babesia microti* (LC005753, Mongolia, 1667 bp)  *Babesia microti* (MT423327, China, 1702 bp)  *Babesia microti* (PP087232, China, 1667 bp)  *Babesia microti* (AF231348, USA, 1705 bp)  *Babesia microti* (AF231348, USA,1705 bp)  *Babesia microti* (LC005769, Mongolia, 1667 bp)  Babesia microti (LC127369, Japan, 1577 bp)  *Babesia microti* (LC127370, Japan, 1552 bp)  *Babesia microti* (MK609547, USA, 2573 bp) |
