# Supplementary material for: A survey for piroplasmids in questing Ixodes fuscipes ticks reveals undescribed Babesia lineages in Uruguay
Source: Parasit Vectors. 2025 Jun 18;18:225. doi: 10.1186/s13071-025-06866-0 (PMC12175381; doi:10.1186/s13071-025-06866-0)
Supplement: Supplementary file 4 — Additional File 4: Supplementary Table S4. BLASTn results of COI sequences of Babesia odocoilei-like obtained in this study. [file 13071_2025_6866_MOESM4_ESM.docx]

**Additional file 4: Table S4.** BLASTn results of *COI* sequences of *Babesia odocoilei*-like obtained in this study.

| **Sample ID (location, stage, GenBank acc. number, sequence size)** | **Query coverage %** | **Identity %** | **Gaps** | **E-value** | **Species or genotypes (GenBank acc. number, Country, sequence size)** |
| --- | --- | --- | --- | --- | --- |
| S21IpN3 (VL, nymph, PP886520, 894 bp) | 75% | 97.45% | 0/667 | 0.0 | *Babesia odocoilei* (MG344849, Canada, 927 bp) |
|  | 100% | 93.29% | 0/894 |  | *Babesia* sp. pudui (ON995389, Chile, 972 bp) |
|  | 100 | 93.18% | 0/894 |  | *Babesia* sp. pudui (ON995394, Chile, 993 bp)) |
| S22IpN4 (VL, nymph, PP886519, 891 bp) | 75% | 97.45% | 0/667 | 0.0 | *Babesia odocoilei* (MG344849, Canada, 927 bp) |
|  | 100% | 93.27% | 0/891 |  | *Babesia* sp. pudui (ON995389, Chile, 972 bp) |
|  | 100% | 93.15% | 0/891 |  | *Babesia* sp. pudui (ON995394, Chile, 993 bp) |
| S32IpN18 (AS, nymph, PP886518, 954 bp) | 70% | 97.46% | 0/670 | 0.0 | *Babesia odocoilei* (MG344849, Canada, 927 bp) |
|  | 99% | 93.63% | 0/942 |  | *Babesia* sp. pudui (ON995389, Chile, 972 bp) |
|  | 100% | 93.61% | 0/954 |  | *Babesia* sp. pudui (ON995394, Chile, 993 bp) |
| S36IpN5 (VL, nymph, PP886517, 891 bp) | 75% | 97.60% | 0/667 | 0.0 | *Babesia odocoilei* (MG344849, Canada, 927 bp) |
|  | 100% | 93.38% | 0/891 |  | *Babesia* sp. pudui (ON995389, Chile, 972 bp) |
|  | 100% | 93.27% | 0/891 |  | *Babesia* sp. pudui (ON995394, Chile, 993 bp) |
| S36IpN6 (VL, nymph, PP886516, 891 bp) | 75% | 97.60% | 0/667 | 0.0 | *Babesia odocoilei* (MG344849, Canada, 927 bp) |
|  | 100% | 93.38% | 0/891 |  | *Babesia* sp. pudui (ON995389, Chile, 972 bp) |
|  | 100% | 93.27% | 0/891 |  | *Babesia* sp. pudui (ON995394, Chile, 993 bp) |
| S39IpN17 (GC, nymph, PP886515, 1002 bp) | 72% | 97.50% | 0/721 | 0.0 | *Babesia odocoilei* (MG344849, Canada, 927 bp) |
|  | 97% | 93.72% | 0/972 |  | *Babesia* sp. pudui (ON995389, Chile, 972 bp) |
|  | 100% | 93.69% | 0/999 |  | *Babesia* sp. pudui (ON995393, Chile, 1002 bp) |
| S44IpN66 (AS, nymph, PP886514, 1002 bp) | 72% | 97.36% | 0/721 | 0.0 | *Babesia odocoilei* (MG344849, Canada, 927 bp) |
|  | 97% | 93.52% | 0/972 |  | *Babesia* sp. pudui (ON995389, Chile, 972 bp) |
|  | 100% | 93.49% | 0/999 |  | *Babesia* sp. pudui (ON995393, Chile, 1002 bp) |
